# A review and meta-analysis of the survival rate of adult with osteonecrosis of the femoral head treated with transtrochanteric rotational osteotomy

**DOI:** 10.1097/MD.0000000000031777

**Published:** 2022-11-25

**Authors:** Yong Xu, Ping Zeng

**Affiliations:** a Graduate School of Guangxi University of Chinese Medicine, Nanning, China; b Department of Orthopedics, The First Affiliated Hospital of Guangxi University of Chinese Medicine, Nanning, China.

**Keywords:** META analysis, ONFH, osteonecrosis of the femoral head, review, transtrochanteric rotational osteotomy, TRO

## Abstract

Osteonecrosis of the femoral head (ONFH) is a progressive disease, and transtrochanteric rotational osteotomy (TRO) is a promising option to treat hip preservation. Compared with outcomes of non-Asian patients, those of Asian patients are different. Once ONFH has progressed to the stage of collapse, total hip arthroplasty (THA) is an unavoidable surgical procedure. However, patients treated with TRO still have a certain rate of femoral head collapse. A meta-analysis was done to comprehensively understand the outcome of TRO surgery for ONFH, in which conversion to THA was used as an endpoint event or the survival rate of patients with ONFH after TRO surgery as an indicator.

**Methods:**

We retrieved electronic databases from the inception of the study until June 2022, using the survival rate after TRO surgery or that after conversion to the endpoint event of THA as the effect indicator. The Risk Difference Independent unmatched samples of counting information with 95% confidence intervals (CI) were used to calculate the outcome. Also, subgroup analysis was carried out for Asian and non-Asian patients. Heterogeneity and publication bias analyses were also done for the included studies

**Results:**

We pooled 19 studies, all of which were about applying TRO procedure for ONFH. There were 15 cohort studies, 4 case-control studies, and no randomized controlled studies. Based on the information extracted from the reported above (we extracted the relevant independent effect values separately for the case-control studies), this meta-analysis was performed based on a fixed-effect model, and META analysis was performed for an independent unpaired group of the samples. The total hip survival rate after TRO in ONFH was 0.58(95% CI = 0.45–0.72), The survival rate for Asians was: 0.68 (95% CI = 0.51–0.85) and for non-Asians was: 0.41 (95% CI = 0.17–0.64), respectively.

**Conclusions:**

The application of TRO surgery in ONFH can effectively relieve patients’ symptoms and they enjoy n a high survival rate, especially for Asian patients. This makes it a promising surgical technique.

## 1. Introduction

Osteonecrosis of femoral head (ONFH) is a progressive disease with a high incidence of disability. The incidence of ONFH in the United States can reach 20,000 per year,^[1]^ and the annual incidence of ONFH in South Korea increased from 9870 in 2002 to 18,691 in 2006.^[2]^ More than 8 million patients were diagnosed with ONFH in China in 2017.^[3]^ To treat the disease, many surgical procedures have been proposed in the late stages of collapse or osteoarthritis of the hip (Ficat III and IV stages).^[4]^ However, none of them seems to bring a good clinical outcome. In terms of prognosis, only one treatment option is available if the diseased femoral head collapses, namely total hip arthroplasty (THA). This is because this procedure improves quality of life and mobility. However, in the population of young patients, the satisfaction rate after 5 years of this surgery is not high.^[5]^ Young patients of this surgery must undergo several other replacements during their long-term life, which will be potentially harmful and financially burdensome for them.^[6]^ The results of revision in terms of functionality and quality of life are much worse than those of initial THA.^[7]^ In addition, many young people with osteonecrosis suffer from many complications after THA, such as loosening of the prosthesis, sagging, wear and loss of polymer liner fragments.^[8]^ Unfortunately, most of ONFH patients happens to be the younger population.^[9,10]^ Therefore, patients could be spared from eventual THA, or at least delay the process of undergoing the procedure if the collapse of the femoral head necrosis in young patients could be effectively avoided. Though there is still no “ideal procedure” to prevent the femoral head from collapsing in the early stages of osteonecrosis, joint-preserving procedures are worth considering for young patients under the age of 20 with osteonecrosis.^[11]^ Sugioka^[12]^ first described the use of a transtrochanteric rotational osteotomy (TRO) to protect the femoral head against secondary osteoarthritis in young patients with osteonecrosis in 1978. Several Japanese studies^[13]^ have shown the favorable results of this treatment. However, the clinical outcomes in Europe and the United States were not satisfactory^[13,14]^: the process of osteoarthritis was not delayed and most patients eventually had to undergo artificial THA. Finally, the authors had to abandon this technique after some resistance: 13 hips were operated by the original technique of Sugioka and 12 ones had to end up with hip arthroplasty in a time of up to 7 years. Analyzing the causes, the authors^[14,15]^ concluded that damage to the blood vessels during the rotational osteotomy was responsible for the poor outcome of the surgical procedure. Another study,^[14,15]^ found that almost all necrotic segments that were not fully rotated out of the weight-bearing area had to receive THA after TRO, indicating that inadequate rotation of the necrotic segment after osteotomy also caused the surgical failure. Given the technical difficulties and the inevitability of intraoperative vascular damage, TRO was abandoned by European and American surgeons. Nevertheless, the procedure is still frequently performed in Japan. Japanese scholars^[16]^ believe that it should be the option for young patients without severe femoral head collapse as a joint-preserving procedure, because they have a stronger capacity for bone remodeling,^[17]^ such as TRO and transtrochanteric curve varus osteotomy. To ensure excellent clinical outcomes of this procedure, patients should be appropriately selected, the procedure should be accurately performed and proper postoperative rehabilitation should be excellently provided.^[18]^ Therefore, a meta-analysis was done to evaluate the effect of TRO surgery for ONFH. Hip survival rate after TRO (around 3–10 years, with THA as the endpoint of the event) was calculated as an effect indicator, and its causes were analyzed for an overview analysis of the relevant literature.^[18]^ Because we present a reviewing article, there is no need for an ethics committee or institutional review board to approve the study.

## 2. Methods

### 2.1. Literature search

**Figure 1.**
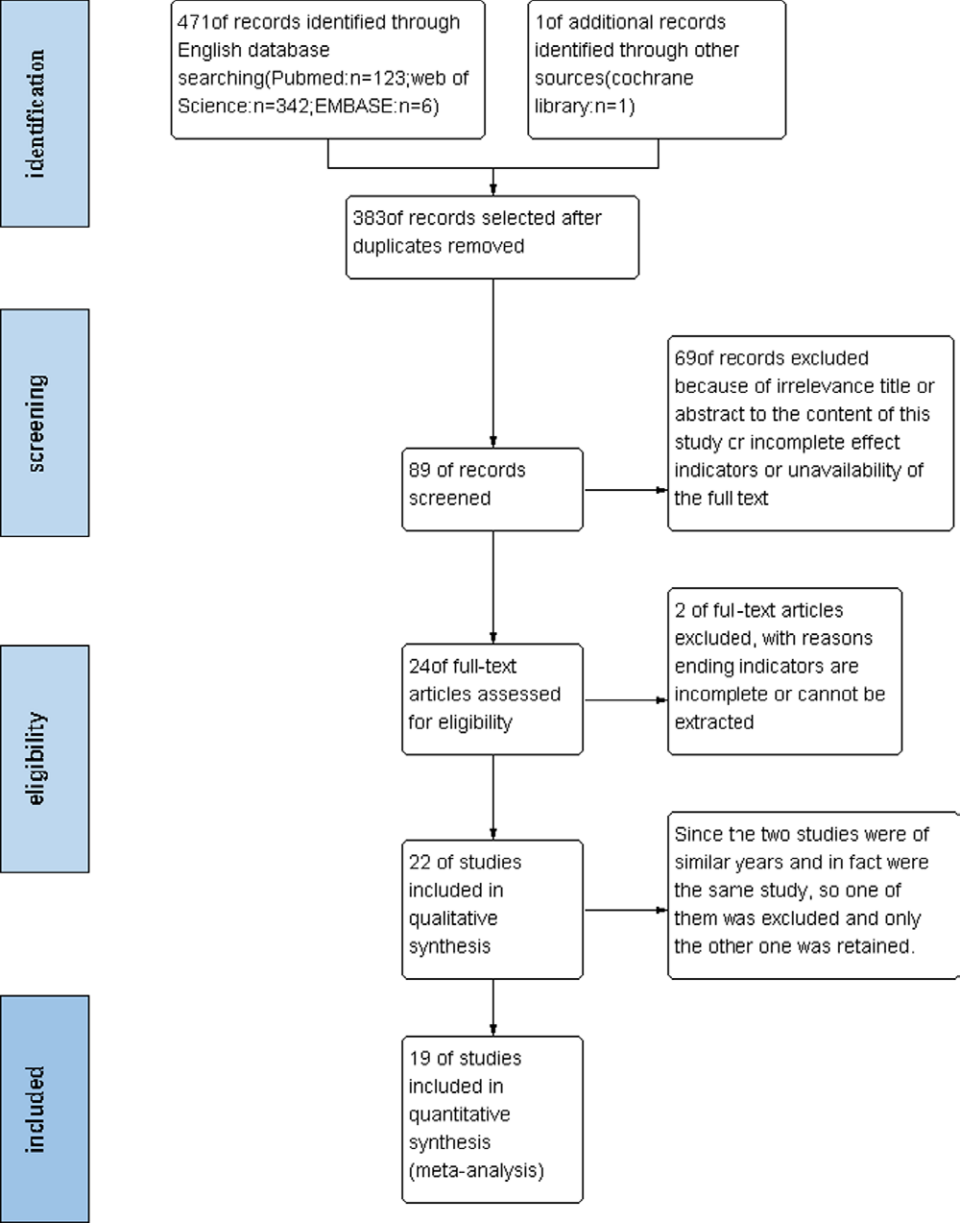
Flow diagram of the study selection procedure.

Electronic databases including PubMed, Embase, the Cochrane Library, and the Web of Science were selected for searching the information from the date of inception of the study to June 2022. The searching strategy for PubMed was described in Table 1. The language was limited to English. We first read the titles and abstracts of the literature and screened out inappropriate literature. Then we further imported eligible studies into EndNote 20 software to screen out duplicate literature removing a part of it. Finally, the obtained literature was searched for the full text for META analysis. Edified trials into Science Citation Index were conducted to identify articles that quoted the original study. Our search was done by two independent researchers (P.Z. and Y.X.). When the search results were inconsistent, a third person (BL.S.) would decide the result. The study selection process strictly followed the PRISMA flowchart (Fig. 1).^[19]^

**Table 1:** Literature search strategies in PUBMED.

((((((((((Femur Head Necrosis[MeSH Terms]) OR (Femur Head Necroses[Title/Abstract])) OR (Head Necrosis, Femur[Title/Abstract])) OR (Necrosis, Femur Head[Title/Abstract])) OR (Aseptic Necrosis of Femur Head[Title/Abstract])) OR (Necrosis, Aseptic, of Femur Head[Title/Abstract])) OR (Necrosis, Avascular, of Femur Head[Title/Abstract])) OR (Ischemic Necrosis Of Femoral Head[Title/Abstract])) OR (Femoral Head, Avascular Necrosis Of[Title/Abstract])) OR (Avascular Necrosis Of Femoral Head, Primary[Title/Abstract])) OR (Avascular Necrosis of Femur Head[Title/Abstract]) AND (((((((Osteotomy[MeSH Terms]) OR (Osteotomies[Title/Abstract])) OR (femur intertrochanteric osteotomy[Title/Abstract])) OR (femoral osteotomy[Title/Abstract])) OR (Intertrochanteric osteotomy[Title/Abstract])) OR (transtrochanteric rotational osteotomy[Title/Abstract])) OR (transtrochanteric osteotomy[Title/Abstract])) OR (Sugioka’s osteotomy[Title/Abstract]) AND
((((((((((((randomized controlled trial[Publication Type]) OR (controlled clinical trial[Publication Type])) OR (randomized[Title/Abstract])) OR (Placebo[Title/Abstract])) OR (randomly[Title/Abstract])) OR (trial[Title/Abstract])) OR (groups[Title/Abstract])) OR (random allocation[Title/Abstract])) OR (double-blind[Title/Abstract])) OR (single-blind[Title/Abstract])) OR (clinical trial*[Title/Abstract])) OR (RCT[Title/Abstract])) OR (Random*[Title/Abstract]

### 2.2. Selection criteria

Adult patients diagnosed with ONFH by clinical signs, symptoms, and imaging data were selected. They underwent surgical management with an TRO. Studies in which Kaplan–Meier survival curves or rate were extractable or in the original text provided data on the endpoint incident converted to an artificial THA were carried out.

The articles were excluded according to the following criteria: reviews, editorials, letters to editors, animal experiments, or surgical techniques, or children’s patients; the studies which did not contain the hip survival rate after TRO or the THA coversion rate or number; and the studies which were not for TRO based on the original technique described by Sugioka.

### 2.3. Data extraction

The data obtained in the original literature were extracted into an Excel sheet to extract the data. We extracted information about patient’s demographic profile characteristics, first author name of the study, nation, and year of publication, study type, total number of patients in the experiments, intervention method, stage of ONFH, number of cases of postoperative disease advancing to THA surgery, and postoperative survival rate in each study. This process was also done by two independent researchers (Z.P. and Y.X.) and finally based on consensus for aggregation. In case of inconsistency, a third individual (BL.S.) decided the result. See Table 2.

**Table 2:** Characteristics of studies included in the meta-analysis.

Study	Author	Year	Country	Study design	Staging	Intervention	THA events (hips)	Total number (hips)	Survival rate (>3 yr)	Follow months
^[44]^	Y. K. Lee	2017	Korea	Case-control	Ficat IIB, III	TRO vs TCVO	–	91	0.832	63
^[11]^	D. Morita	2017	Japan	Cohort study	Ficat II, III	TRO	–	111	0.59	60
^[48]^	Yong-Chan Ha	2010	Korea	Cohort study	Ficat II, III	TRO	14	127	0.560	110
^[49]^	Koichiro Kawano	2020	Japan	Case-control	Ficat III	TRO vs conservative treatment	–	54	0.504	218.4
^[50]^	Y. Hasegawa	1999	Japan	Case-control	Ficat I, II, III	ARO/PRO/TRO/	–	77	0.61	84
^[26]^	S. Sakano	2004	Japan	Case-control	–	ARO/VWO	2	20	0.71	48
^[51]^	K. Sonoda	2015	Japan	Case-control	Ficat II, III	ARO/PRO	6	28	0.785	147.6
^[25]^	M. T	1993	USA	Cohort study	Ficat III, IV	TRO	12	18	0.66	60
^[34]^	S. Iwasada	1997	Japan	Cohort study	–	TRO	14	113	0.634	51.3
^[13]^	W. H. C. Rijnen	2005	Netherlands	Cohort study	ARCO2, 3	TRO	5	48	0.560	55.2
^[52]^	A. Michael	1989	Japan	Cohort study	Ficat II, III	TRO	2	20	0.727	63
^[40]^	N. Sugano	1992	Japan	Cohort study	Ficat II, III	TRO/flexion/extention	6	22	0.78	63
^[14]^	P. Grigoris	1996	UK	Cohort study	FicatIII, IV	TRO	14	20	0.30	84
^[53]^	Yusuke Kubo	2016	UK	Cohort study	Ficat III, IV	ARO	14	20	0.30	85.2
^[29]^	Yusuke Osawa	2020	Japan	Case-control	Ficat II, III	CVO VS CVO + BIG	6	47	0.777	120
^[17]^	Michio Hamanishi	2014	Japan	Cohort study	Ficat I, II, III	TRO	1	53	0.962	55
^[18]^	Shin Onodera	2005	Japan	Cohort study	ARCO II, III, IV	TRO	9	38	0.763	48
^[53]^	Y. Kubo	2016	Japan	Cohort study	ARCO II, III, IV	AROund	8	47	0.648	120
^[55]^	W. Schneider	2002	Austria	Cohort study	Steinberg IV, V, VI	TRO/flexion osteotomy	63	115	0.325	7.3

TCVO = transtrochanteric curve varus osteotomy, THA = total hip arthroplasty, TRO = transtrochanteric rotational osteotomy.

### 2.4. Assessment of heterogeneity

The chi-square and *I*^2^ were used to estimate the heterogeneity of the studies. If *P* ≥ .05 and *I*^2^ < 50%., the result indicated that the heterogeneity was relatively small, and a fixed-effect statistical model was used. Otherwise, a random-effect model is preferred. If the heterogeneity was strong, we further did subgroup analysis to understand its origin.

### 2.5. Assessing risk of bias in the included studies

The Newcastle–Ottawa scale was used to perform the assessment of the quality of the included studies. This was the scale used to assess the risk of bias in case-control trials as well as in cohort studies. Finally, any discrepancies in the bias results between studies were resolved by discussion (Table 3).

**Table 3:** Quality assessment of cohort studies.

Study (authors, year)	Representativeness of the exposed cohort	Election of the non exposed cohort	Ascertainment of exposure	Demonstration that outcome of interest was not present at start of study	Comparability of cohorts on the basis of the design or analysis	Assessment of outcome	Was follow- up long enough for outcomes to	Adequacy of follow up of cohorts
Koichiro Kawano, 2020	Yes	Yes	Yes	Yes	NA	Yes	Yes	Yes
Yong-Chan Ha 2010	Yes	NA	Yes	Yes	NA	Yes	Yes	Yes
D. Morita, 2017	Yes	NA	Yes	Yes	NA	Yes	Yes	Yes
Y. Hasegawa 1999	Yes	NA	Yes	Yes	NA	Yes	Yes	Yes
Y.-C. Ha, 2010	Yes	NA	Yes	Yes	NA	Yes	Yes	Yes
Y. Kubo 2016	Yes	NA	Yes	Yes	NA	Yes	Yes	Yes
S. Sakano, 2003	Yes	NA	Yes	Yes	NA	Yes	Yes	Yes
Michael A, 1989	Yes	NA	NA	Yes	NA	Yes	Yes	Yes
Shin Onodera 2005,	Yes	NA	Yes	Yes	NA	Yes	Yes	Yes
N. Sugano, 1992	Yes	NA	Yes	Yes	NA	Yes	Yes	Yes
K. Sonoda, 2015								
Y. K. Lee 2017	Yes	NA	Yes	Yes	NA	Yes	Yes	Yes
S. Iwasada, 1997	Yes	NA	Yes	Yes	NA	Yes	Yes	Yes
YusukeOsawa, 2020	Yes	NA	Yes	Yes	NA	Yes	Yes	Yes
Michio Hamanishi , 2014	Yes	NA	Yes	Yes	NA	Yes	Yes	Yes
P. Grigoris, 1996	Yes	NA	Yes	Yes	NA	Yes	Yes	Yes
W. Schneider2002	Yes	NA	Yes	Yes	NA	Yes	Yes	Yes
W. H. C. Rijnen, 2005	Yes	NA	Yes	Yes	NA	Yes	Yes	Yes
M. T. Dean, 1993	Yes	NA	Yes	Yes	NA	Yes	Yes	Yes

Publication bias was evaluated when generating funnel plots were generated and examined for any obvious visual asymmetry.

The Grading of Recommendations Assessment, Development and Evaluation^[20]^ system was adopted to assess the overall quality of the evidence.

### 2.6. Binary data of sensitivity analysis

When appropriate, we excluded studies with significant heterogeneity used for sensitivity analysis to understand the specific cause*s*.

### 2.7. Statistical analysis

The Revman 5.4.1 software (provided by the Cochrane Collaboration,) was used for the statistical analysis. we calculated RD (risk difference) and 95% confidence limits for dichotomous outcomes, (hip survival rate after TRO and THA surgery conversion rate). The data including hip survival rate or provided in all the included studies were considered as noncomparative binary variables, and the effect indicator with their standard errors were calculated whit the method according to the statistical method provided by Chen et al.^[21,22]^ We conducted meta-analyses with a fixed-effect model. Where there was statistical evidence of heterogeneity (a chi squared test with *P* < .05 or an *I*-squared test with a percentage of the variability in effect estimates > 50%), we used a randomized-effect model. *P* < .05 was considered to be statistically significant. Results were graphically represented by forest plots. Of the included studies, 15 were cohort studies without control groups, and 4 were case-control studies with dissimilar control groups. Therefore, the following parameters were used: P, the hip survival rate after TRO; standard errors; X, and the number of hip survival patient.

## 3. Results

### 3.1. Search results

After searching the databases, reviewing titles and abstracts, removing duplicates, and following the criteria for inclusion, we included a total of 19 studies in our META analysis study. Among them, 14 were from Japan and South Korea, and the remaining 5 were from the United Kingdom, the United States, Australia, and the Netherlands. Except 6 studies were case-control studies, all the rest were cohort studies. Since the control groups of the case-control studies were not uniform, the relevant data regarding osteotomy were extracted separately from all these studies.

### 3.2. Assessing methodologic quality of the included studies and the public bias

**Figure 2.**
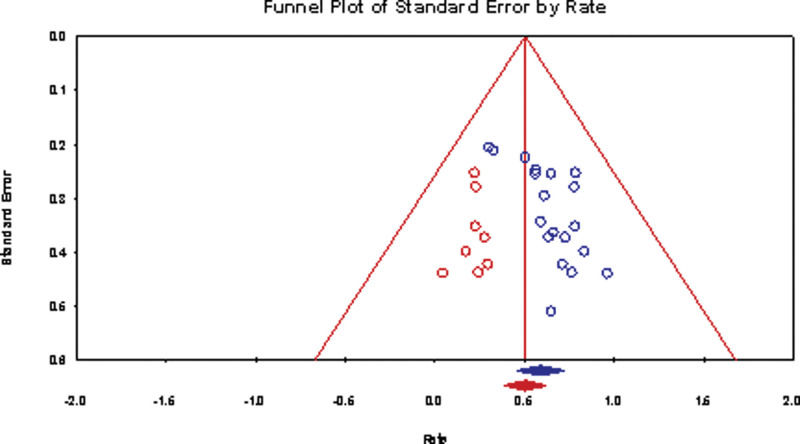
Funnel plot for public bias analysis.

From the funnel plot, the studies we included had some publication bias. The Newcastle–Ottawa scale and Grading of Recommendations Assessment, Development and Evaluation were used for methodological assessments, and the specific assessment scales for the 19 included studies were shown in Figure 2, Tables 3 and 4.

**Table 4:** Methodological assessments by the GRADE software.

Quality assessment							No of patients		Effect		Quality	Importance
No of studies	Design	Risk of bias	Inconsistency	Indirectness	Imprecision	Other considerations	Meta-analysis of uncontrolled categorical data	Control	Relative (95% CI)	Absolute		
p, SE (follow-up mean 12 yr)												
19	Observational studies	No serious risk of bias	No serious inconsistency	Serious^*^	No serious imprecision	None	– 1069	–0%	See comment	– –	⊕ΟΟ LOW	IMPORTANT
p, SE – Asia												
14	Observational studies	No serious risk of bias	No serious inconsistency	Serious^*^	No serious imprecision	Strong association^*^	801	0%	See comment	– –	⊕⊕ΟΟ LOW	IMPORTANT
p, SE – Nonasia												
5	Observational studies	Little serious*	Serious*	No serious indirectness	No serious imprecision	None	268	– 0%	See comment	– –	⊕ΟΟ LOW	IMPORTANT

Author(s): yong xu.

Date: Jun 27, 2022.

Question: META analysis for femoral head necrosis.

Settings: none.

Bibliography: osteotomy for femoral head necrosis. Cochrane Database of Systematic Reviews [Year], Issue [Issue].

CI = confidence intervals, GRADE = The Grading of Recommendations Assessment, Development and Evaluation, SE = standard errors.

* No explanation was provided.

### 3.3. Hip survival rate after TRO, with conversion to THA as the endpoint

**Figure 3.**
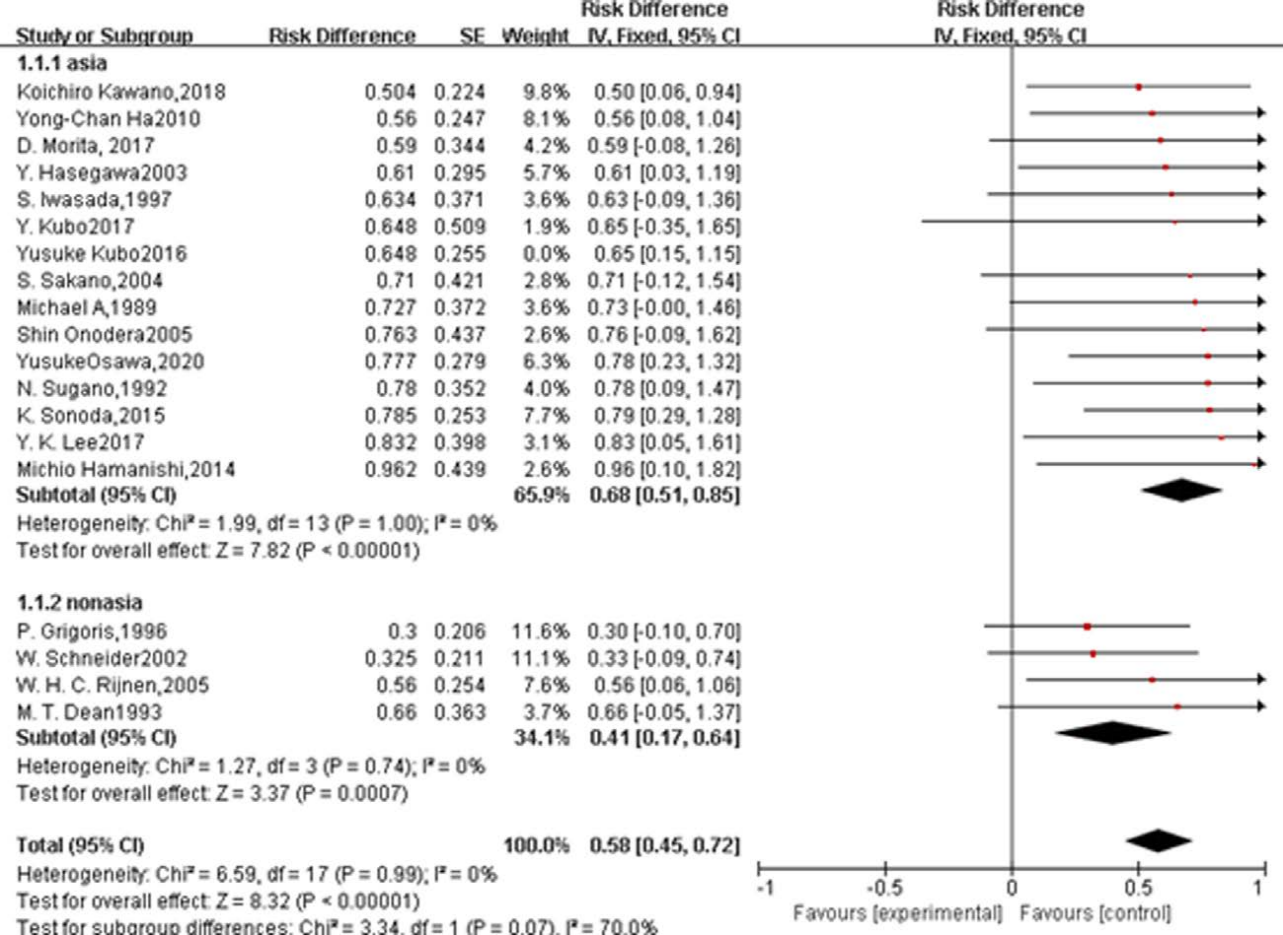
Forest plot for studies and subgroup studies. CI = confidence intervals, SE = standard errors.

A total of 19 studies with a number of 1069 joints undergoing transtrochanteric rotational osteotomies for ONFH were conducted. Heterogeneity was: Chi^2^ = 6.59, df = 17 (*P* = .99); *I*^2^ = 0%, The overall results showed little heterogeneity, so the fixed-effect model was used for the analysis of association. Test for the overall effect: *Z* = 8.32 (*P* < .00001) The overall effective survival rate was 0.58%, with 95% CI (45–72%) (Fig. 3).

### 3.4. Subgroup analysis

Further subgroup analysis was done, and all the studies were divided into Asian and non-Asian subgroups according to the literature. The results showed a statistical difference between the two groups, Test for subgroup differences were: Asian group: Chi^2^ =1.99, df = 13 (*P* = 1.00). The survival rate of the Asian group was 0.68 [0.51, 0.85], while that of the non-Asian group was 0.41 [0.17, 0.64]. Test for subgroup difference was: Chi^2^ = 3.34, df = 1 (*P* = .0007); *I*^2^ = 70%.

## 4. Discussion

TRO for ONFH was first reported in 1978 in an article by Sugioka in Japan12.Theoretically, osteotomy is an ideal surgical procedure to treat ONFH. Its basic principle is to free the necrotic part of the diseased femoral head from the weight-bearing space and replace the damaged bone tissue by normal articular cartilage by the operation of TRO (TRO)^[23]^ In the early stages of femoral head necrosis, TRO is considered to be a very effective surgical procedure to treat hip preservation. This is especially true in Japan.^[24]^ Although the results of this procedure were relatively positive in Japan, surgeons in Europe and the United States have not achieved the same desired results when applying this surgical technique. The failure of this procedure was marked by the artificial THA treatment of patient. As a result, a meta-analysis was performed using the survival rate from the time when a TRO was received until the end event of artificial THA as an indicator in this paper. When THA surgery was the end event, the persistent necrosis and secondary collapse of the femoral head of ONFH treated with TRO surgery was the main relevant pathological process. How to avoid the consequential osteonecrosis and secondary collapse of the surgically treated femoral head to prevent the eventual THA is the current research hotspot and a difficult issue in this surgery.

Undoubtedly, disrupting the blood supply to the femoral head after osteotomy is a leading cause to progressive necrosis and the secondary collapse.^[25]^ Some scholar^[26]^ have examined the blood supply to the femoral head after TRO with technetium 99m bone scan, finding that inevitably all postoperative blood supply to the femoral head was disrupted. However, this disruption may also have been prior to the surgery. Therefore, selective radionuclide angiography has been advocated^[27]^ to identify the preoperative vascular status and preserve the arteries prior to TRO. Besides, MRI has been used^[28]^ to assess the arterial circulation in preoperative cases of femoral head necrosis: when the blood supply is inadequate in the quadratus femoris or is disrupted during surgery, the arterial circulation may be disrupted. The patient will be at a greater risk of postoperative femoral head collapse. So, it is suggested in the operation procedure that the short external rotators should be protected from being disturbed by the quadratus femoris.^[12]^ Postoperative testing of the circulatory status is generally considered to be helpful in predicting the outcome of the surgery with a bone scan in 3 weeks.^[29]^

In addition to blood supply components, many factors are associated with increased incidence of persistent necrosis and secondary collapse of the femoral head after TRO. One study^[30]^ used a Cox risk-proportional model analysis to examine the risk of collapse of the femoral head after TRO. The study found that the age of the preoperative patient was >40 years, body mass index >24, stage of osteonecrosis above Ficat stage IIIB, and size of the necrosis >36% of the entire articular surface area. These were highly associated with the risk of postoperative collapse among multiple variables. In another study,^[29]^ lower preoperative for The Harris Hip Score, severe degree of necrotic lesion and intact lower postoperative were also found to be highly correlated with the progressive collapse of TRO. Their study further revealed that the risk of collapse was also closely correlated when the intact area of the posterior femoral head was less than 36% in lateral segment. This finding has been confirmed by other studies.^[12,29,31]^ Research^[32]^ have further confirmed that the risk of collapse is much lower when more than one-third of the entire articular surface of the hip possibly remains intact on the lateral radiograph of the femoral head postoperatively. This rate is not uniform, ranging from 34%^[33]^ to 50%^[34]^or 45%.^[35]^ The exact percentage of necrosis associated with the collapse is also controversial. However, the risk of collapse is low if the posterior 1/3 of the femoral head is intake preoperatively in general. In fact, no matter what the size of the necrotic area or of the proportion of intact area under the weight-bearing zone after surgery are, secondary hip instability is the main pathological mechanism in analyzing collapse-related factors.^[35]^ Pathological studies have found that the pathological process of necrosis does not stop^[36]^ though the necrotic femoral head is removed from the weight-bearing zone. On this basis, the instability of the hip joint is accelerated and secondary to osteoporosis. While the articular cartilage under the new weight-bearing zone after rotation is healthy, the newly established healthy zone is insufficient to bear the weight, easily leading to fatigue fractures. The healthy zone of the “beak-like” structure after rotational osteotomy is relatively thin and beak-shaped, and the risk associated with collapse is reduced only when the proportion of the healthy articular cartilage in the rotated acetabulum is more than 50% of the acetabular area.^[30]^ In contrast,^[37]^ three-dimensional finite-element studies have found that the risk of collapse is higher in femoral heads with a wider necrotic zone due to high local stress on the zone surface. On the contrary, femoral heads with a narrow necrotic zone tend to expand rather than collapse due to high local stress on the interface between the necrotic zone and the healthy one. One study^[29]^ has specifically investigated the relationship between the collapse height followed by breakdown to conclude that when a volume of collapse height was greater than 2.98 mm, the risk of secondary collapse subsequently increased.

It is believed that the occurrence of postoperative femoral head collapse of patients is not related to choosing the intraoperative approach or method of internal fixation.^[25,38]^ Although the application of stainless steel cannulated screws, titanium screws and a special dynamic hip screw were reported in Sugioka’s literature^,[39]^ he changed the approach of internal fixation to AO plates in his subsequent study. This was believed to counteract counter-rotational forces and promote fracture healing.^[38]^ It is convinced that gentle manipulation and reliable fixation during the procedure will help prevent possible postoperative collapse of the articular cartilage.^[40]^ Pathologically,^[41]^ only incomplete regeneration of the transposed osteonecrosis lesion with cysts, sclerosis and fragmentation occurred after osteotomy management, and only partially incomplete repair of the trabeculae was observed in the femoral head in one third of the patients after internal fixation. This also confirms that the surgical approach of internal fixation is not correlated with femoral head collapse.

**Figure 4.**
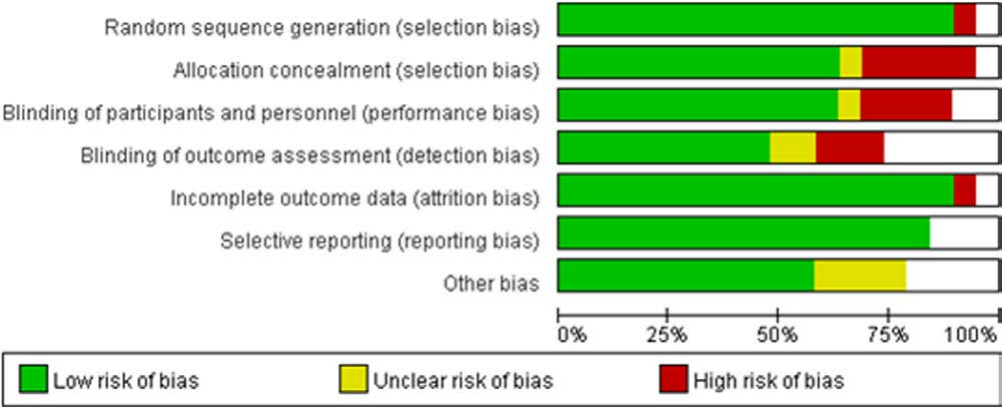
Illustration of TRO and TCVO procedures. TCVO = transtrochanteric curve varus osteotomy, TRO = transtrochanteric rotational osteotomy.

Since the posterior column artery which branches out from the medial femoral circumflex artery moves medially and into a relaxed position, it is safe to rotate posteriorly and is dangerous to rotate forward during the osteotomy.^[18]^ The result is opposite to the three-dimensional finite-element research.^[29]^ One retrospective comparison^[33]^ revealed that the operation time of transtrochanteric curve varus osteotomy Figure 4 is shorter and less bleeding, with lower incidence of osteophyte formation and lower rate of secondary collapse. Atsumi and Kuroki^[42]^ modified the TRO approach by flexing the rotational femoral head forward based on external and varus rotation. They believed that this modified procedure could improve the blood supply to the femoral head after the surgery. If the degree of rotation is not sufficient, the risk of postoperative necrosis and secondary collapse is much higher as the necrotic portion cannot be effectively rotated out of the weight-bearing area. This is correlated with the poor results when this procedure was initially applied by European and American surgeons.^[43]^ The indications^[44]^ for TRO surgery are that the patient’s age is below 40 years, body mass index less than 24 kg/m^2^, ischemic necrosis of the femoral head in an early stage, i.e. before collapse, and the patient should have modest active bone mass. All complications^[40]^ after TRO surgery focused on the femoral neck are the following: nonunion of the fracture, further collapse of the femoral head, and aggressive osteoarthritis.

Although Sugioka^[45]^ reported a low rate of postoperative fracture and nonunion rates, this might be explained by intensive postoperative care and prolonged bed rest with this surgical approach. Ethnicity-related factors have been suggested to account^[25]^ for the different surgical outcomes of Japanese versus European and American patients undergoing TRO: Japanese patients have a more relaxed posterior hip capsule and can hence rotate forward at a greater angle without damaging the blood supply arteries to the femoral head.^[25,46]^ Of course, it is also possible that there are race-dependent differences in weight, height, and anatomy of the hip capsule between Asian and non-Asian patients, ultimately affecting the outcome of osteotomies.^[30,46,47]^ European orthopaedical surgeons believe that the reasons for such differences may also include inappropriate patient selection, inadequate surgical technique, or incorrect internal fixation methods.^[13,39]^ However, Japanese scholars believe that this osteotomy is only suitable for patients with ARCO stage I and II.^[16]^

**Figure 5.**
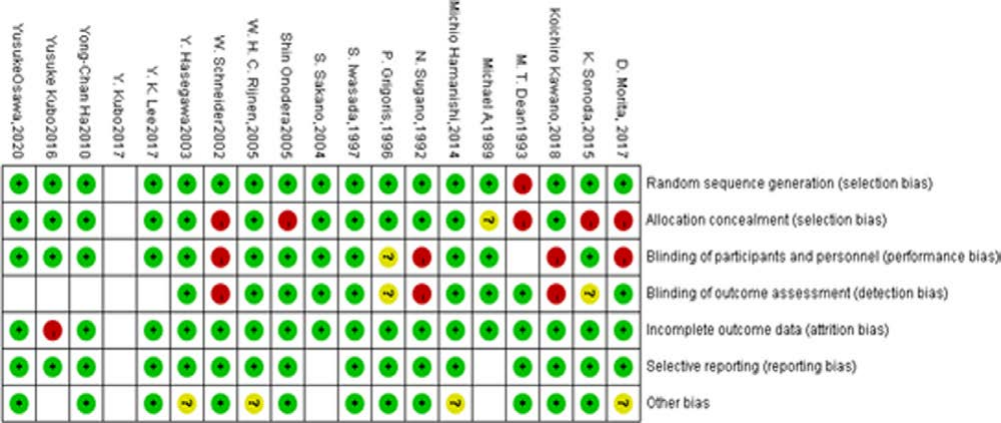
Risk of bias graph: review authors’ judgements about each risk of bias item presented as percentages across all included studies.

**Figure 6.**
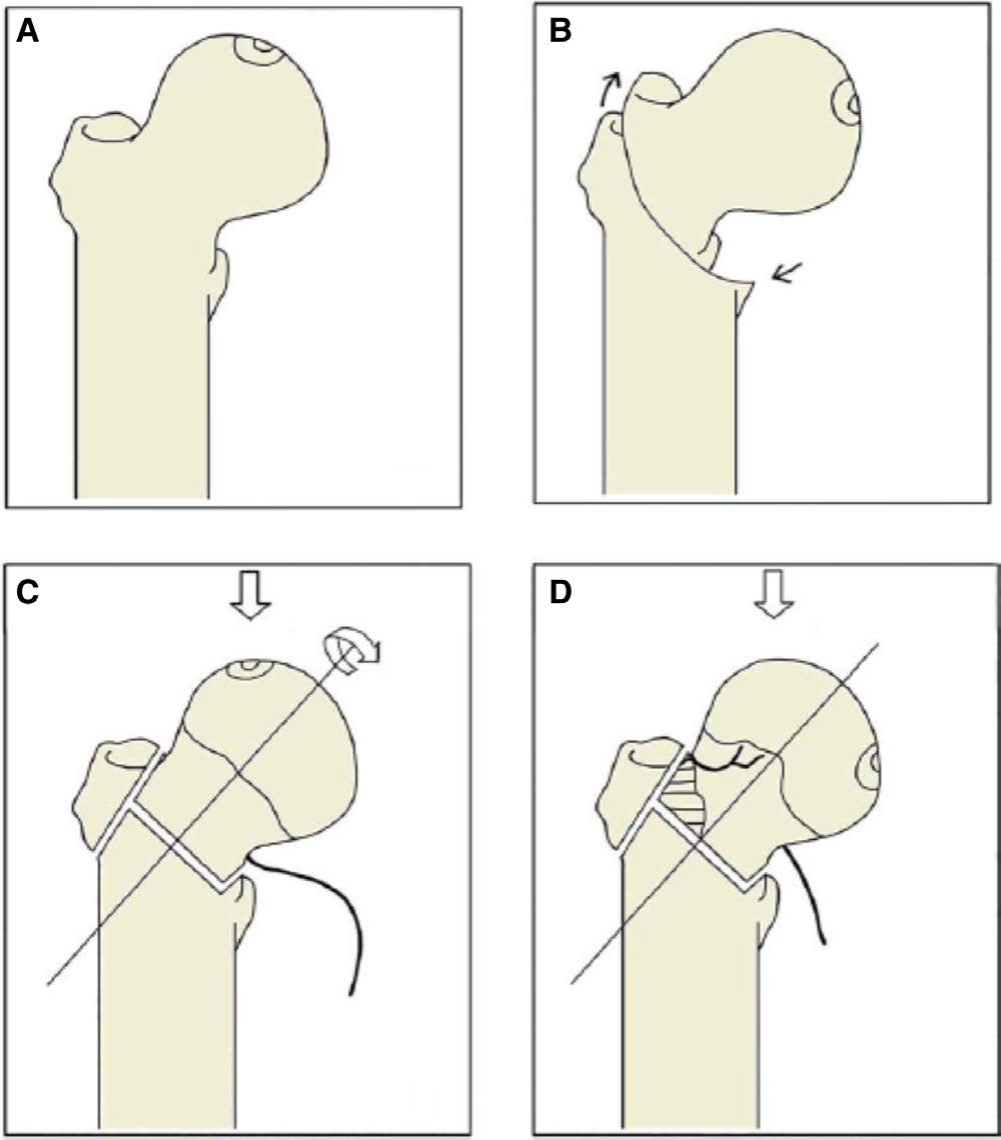
Risk of bias summary: review authors’ judgements about each risk of bias item for each included study.

Since there are few relevant RCT studies, our study found some heterogeneity in the relevant studies. Figures 5 and 6, although our studies were all observational studies and the persuasiveness of the evidence was low, the grading showed that all were significant. See Table 4 However, there are still some defects in our study. Since they are all observational cohort studies, there are no RCT studies available at all. Besides, the sample size is small. There is a large heterogeneity among studies, especially the data of non-Asian patients are not large enough and the evidence is not convincing enough. However, it is the first META analysis about TRO for ONFH with a great potential for future improvement.

In conclusion, our META analysis found that TRO procedure for ONFH is an early alternative and a good hip preservation procedure that can effectively reduce the progression of the patient’s disease, relieve the symptoms, and even avoid the eventual artificial THA. The overall survival rate is 58% (45–72%), with a higher rate for Asian patients: 68% (51–85%) and 41% (17–64%) for non-Asian patients. The subgroup analysis shows a statistically significant difference (*P* = .0007), for which the exact reasons need to be further investigated.

## Author contributions

**Conceptualization:** Yong Xu.

**Data curation:** Yong Xu.

**Formal analysis:** Yong Xu.

**Investigation:** Yong Xu.

**Methodology:** Yong Xu.

**Project administration:** Yong Xu.

**Resources:** Yong Xu.

**Supervision:** Yong Xu, Ping Zeng.

**Writing – original draft:** Yong Xu.

## References

[1] Koo K Kim R Ko G. Preventing collapse in early osteonecrosis of the femoral head. A randomised clinical trial of core decompression. J Bone Joint Surg Br. 1995;77:870–4. 7593097

[2] Kang JS Park S Song JH. Prevalence of osteonecrosis of the femoral head: a nationwide epidemiologic analysis in Korea. J Arthroplasty. 2009;24:1178–83. 19640674 10.1016/j.arth.2009.05.022

[3] Hao Y Guo H Xu Z. The meta-analysis of the potential role of extracorporeal shockwave therapy in osteonecrosis of the femoral head. J Orthop Surg Res. 2018;13:166. 29970103 10.1186/s13018-018-0861-7 PMC6030764

[4] Motomura G Yamamoto T Suenaga K. Long-term outcome of transtrochanteric anterior rotational osteotomy for osteonecrosis of the femoral head in patients with systemic lupus erythematosus. Lupus. 2010;19:860–5. 20305049 10.1177/0961203310361896

[5] Mei XY Gong YJ Safir O. Long-term outcomes of total hip arthroplasty in patients younger than 55 years: a systematic review of the contemporary literature. Can J Surg. 2019;62:249–58. 31348632 10.1503/cjs.013118 PMC6660275

[6] Tan B Li W Zeng P. Epidemiological study based on china osteonecrosis of the femoral head database. Orthop Surg. 2021;13:153–60. 33347709 10.1111/os.12857 PMC7862166

[7] Utsunomiya T Motomura G Ikemura S. The results of total hip arthroplasty after sugioka transtrochanteric anterior rotational osteotomy for osteonecrosis. J Arthroplasty. 2017;32:2768–73. 28529111 10.1016/j.arth.2017.04.034

[8] Hartley WT McAuley JP Culpepper WJ. Osteonecrosis of the femoral head treated with cementless total hip arthroplasty. J Bone Joint Surg Am. 2000;82:1408–13. 11057468 10.2106/00004623-200010000-00006

[9] Ikeuchi KK Hasegawa Y Seki T. Epidemiology of nontraumatic osteonecrosis of the femoral head in Japan. Mod Rheumatol. 2015;25:278–81. 25036228 10.3109/14397595.2014.932038

[10] Mont M Hungerfold D. Current concept review: nontraumatic avascular necrosis of the femoral head. J Bone Joint Surg. 1995;459:74. 10.2106/00004623-199503000-00018 7890797

[11] Ha Y-C Kim HJ Kim S-Y. Effects of age and body mass index on the results of transtrochanteric rotational osteotomy for femoral head osteonecrosis. J Bone Joint Surg. 2011;93:75–84. 21411688 10.2106/JBJS.J.01215

[12] Sugioka Y. Transtrochanteric anterior rotational osteotomy of the femoral head in the treatment of osteonecrosis affecting the hip: a new osteotomy operation. Clin Orthop Relat Res. 1978;130:191–201. 639389

[13] Rijnen WHC Gardeniers JWM Westrek BLM. Sugioka’s osteotomy for femoral-head necrosis in Young Caucasians. Int Orthop. 2005;29:140–4. 15830239 10.1007/s00264-005-0639-5 PMC3456887

[14] Grigoris P Safran M Brown I. Long-term results of transtrochanteric rotational osteotomy for femoral head osteonecrosis. Arch Orthop Trauma Surg. 1996;115:127–30. 8861575 10.1007/BF00434538

[15] Courpied JP. Trans-trochanteric rotation osteotomy for femoral head necrosis. Long-term results. Rev Chir Orthop Reparatrice Appar Mot. 1994;80:694–701. 7638398

[16] Satoshi Ikemura M Takuaki Y Yasuharu N. Transtrochanteric anterior rotational osteotomy for osteonecrosis of the femoral head in patients 20 years or younger. J Pediatr Orthop. 2009;29:4. 10.1097/BPO.0b013e31819bc746 19305269

[17] Hamanishi M Yasunaga Y Yamasaki T. The clinical and radiographic results of intertrochanteric curved varus osteotomy for idiopathic osteonecrosis of the femoral head. Arch Orthop Trauma Surg. 2014;134:305–10. 24394984 10.1007/s00402-013-1919-y

[18] Onodera S Majima T Abe Y. Transtrochanteric rotational osteotomy for osteonecrosis of the femoral head: relation between radiographic features and secondary collapse. J Orthop Sci. 2005;10:367–73. 16075168 10.1007/s00776-005-0906-8

[19] Liberati A Altman DG Tetzlaff J. The PRISMA statement for reporting systematic reviews and meta-analyses of studies that evaluate healthcare interventions: explanation and elaboration. BMJ. 2009;339:b2700. 19622552 10.1136/bmj.b2700 PMC2714672

[20] Guyatt G Oxman AD Akl EA. GRADE guidelines: 1. Introduction-GRADE evidence profiles and summary of findings tables. J Clin Epidemiol. 2011;64:383–94. 21195583 10.1016/j.jclinepi.2010.04.026

[21] Xu YX Ren YZ Zhao ZP. Hip survival rate in the patients with avascular necrosis of femoral head after transtrochanteric rotational osteotomy: a systematic review and meta-analysis. Chin Med J (Engl). 2019;132:2960–71. 31855958 10.1097/CM9.0000000000000562 PMC6964954

[22] Chen Y Du L Geng G. Meta-analysis of uncorrelated dichotomous data in RevMan Software. Chin J Evid Based Med. 2014;14:889–96.

[23] Quaranta M Miranda L Oliva F. Osteotomies for avascular necrosis of the femoral head. Br Med Bull. 2021;137:98–111. 33454780 10.1093/bmb/ldaa044

[24] Sugioka Y Hotokebuchi T Tsutsui H. Transtrochanteric anterior rotational osteotomy for idiopathic and steroid-induced necrosis of the femoral head: indications and long-term results. Clin Orthop Relat Res. 1992;277: 111–20. 1555330

[25] Dean MT Cabanela ME. Transtrochanteric anterior rotational osteotomy for avascular necrosis of the femoral head. Long-term results. J Bone Joint Surg Br. 1993;75:597–601. 8331115 10.1302/0301-620X.75B4.8331115

[26] Hasegawa Y Sakano S Iwase T. Pedicle bone grafting versus transtrochanteric rotational osteotomy for avascular necrosis of the femoral head. J Bone Joint Surg Br. 2003;85:191–8. 12678351 10.1302/0301-620x.85b2.13190

[27] Yasunaga Y Ikuta Y Omoto O. Transtrochanteric rotational osteotomy for osteonecrosis of the femoral head with preoperative superselective angiography. Arch Orthop Trauma Surg. 2000;120:437–40. 10968534 10.1007/s004029900130

[28] Yamamoto T Motomura G Karasuyama K. Results of the sugioka transtrochanteric rotational osteotomy for osteonecrosis: frequency and role of a defect of the quadratus femoris muscle in osteonecrosis progression. Orthop Traumatol. 2016;102:387–90. 10.1016/j.otsr.2016.01.017 26969207

[29] Osawa Y Seki T Okura T. Curved intertrochanteric varus osteotomy vs total hip arthroplasty for osteonecrosis of the femoral head in patients under 50 years old. J Arthroplasty. 2020;35:1600–5. 32063410 10.1016/j.arth.2020.01.026

[30] Ha Y-C Kim HJ Kim S-Y. Effects of age and body mass index on the results of transtrochanteric rotational osteotomy for femoral head osteonecrosis. J Bone Joint Surg. 2010;92:314–21. 20124057 10.2106/JBJS.H.01020

[31] Zhao G Yamamoto T Ikemura S. Clinico-radiological factors affecting the joint space narrowing after transtrochanteric anterior rotational osteotomy for osteonecrosis of the femoral head. J Orthop Sci. 2012;17:390–6. 22580866 10.1007/s00776-012-0238-4

[32] Miyanishi K Noguchi Y Yamamoto T. Prediction of the outcome of transtrochanteric rotational osteotomy for osteonecrosis of the femoral head. J Bone Joint Surg Br. 2000;82:512–6. 10855873 10.1302/0301-620x.82b4.10065

[33] Lee YK Park CH Ha YC. Comparison of surgical parameters and results between curved varus osteotomy and rotational osteotomy for osteonecrosis of the femoral head. Clin Orthop Surg. 2017;9:160–8. 28567217 10.4055/cios.2017.9.2.160 PMC5435653

[34] Iwasada S Hasegawa Y Iwase T. Transtrochanteric rotational osteotomy for osteonecrosis of the femoral head. 43 patients followed for at least 3 years. Arch Orthop Trauma Surg. 1997;116:447–53. 9352037 10.1007/BF00387576

[35] Hiranuma Y Atsumi T Kajiwara T. Evaluation of instability after transtrochanteric anterior rotational osteotomy for nontraumatic osteonecrosis of the femoral head. J Orthop Sci. 2009;14:535–42. 19802664 10.1007/s00776-009-1363-6

[36] Hisatome T Yasunaga Y Takahashi K. Progressive collapse of transposed necrotic area after transtrochanteric rotational osteotomy for osteonecrosis of the femoral head induces osteoarthritic change. Mid-term results of transtrochanteric rotational osteotomy for osteonecrosis of the femoral head. Arch Orthop Trauma Surg. 2003;124:77–81. 14658077 10.1007/s00402-003-0610-0

[37] Lee MS Tai CL Senan V. The effect of necrotic lesion size and rotational degree on the stress reduction in transtrochanteric rotational osteotomy for femoral head osteonecrosis - A three-dimensional finite-element simulation. Clin Biomech. 2006;21:969–76. 10.1016/j.clinbiomech.2006.05.005 16806615

[38] Rotational osteotomy for non-traumatic avascular necrosis of the femoral head. 10.1302/0301-620X.74B5.1527125 1527125

[39] Sugioka Y. Results and indications of transtrochanteric rotational osteotomy. Chirurgie - Mem de l'Acad de Chirurgie. 1987;113:617–23. 3436219

[40] Sugano N Takaoka K Ohzono K. Rotational osteotomy for non-traumatic avascular necrosis of the femoral head. J Bone Joint Sur Series B. 1992;74:734–9. 10.1302/0301-620X.74B5.1527125 1527125

[41] Yamashita A Yamamoto T Jingushi S. Histopathological study of osteonecrosis 19 years after transtrochanteric rotational osteotomy. J Orthop Sci. 2006;11:632–7. 17139474 10.1007/s00776-006-1067-0

[42] Atsumi T Kuroki Y. Modified Sugioka’s osteotomy: more than 130 degrees posterior rotation for osteonecrosis of the femoral head with large lesion. Clin Orthop Relat Res. 1997;334:98–107. 9005901

[43] Hanslik L Scholz J. Transtrochanteric rotational osteotomy for idiopathic avascular necrosis of the femoral head. Z Orthop Ihre Grenzgeb. 1981;119:504–11. 7314824 10.1055/s-2008-1053325

[44] Lee YK Lee B Parvizi J. Which osteotomy for osteonecrosis of the femoral head and which patient for the osteotomy? Clin Orthop Surg. 2019;11:137–41. 31156763 10.4055/cios.2019.11.2.137 PMC6526125

[45] Watabe T Nagoya S Takada J. The transtrochanteric rotational osteotomy of the femoral head for osteoarthritis of the hip joint with subluxation of the femoral head. Hokkaido J Orthop Traumatol. 2004;45:1–4.

[46] Schneider E Ahrendt J Niethard FU. [Long-term results following intertrochanteric varus osteotomy in aseptic femur head necrosis in the adult]. Rofo. 1989;150:402–6. 2539616 10.1055/s-2008-1047046

[47] Kubo Y Motomura G Ikemura S. Factors influencing progressive collapse of the transposed necrotic lesion after transtrochanteric anterior rotational osteotomy for osteonecrosis of the femoral head. Orthop Traumatol. 2017;103:217–22. 10.1016/j.otsr.2016.10.019 28017874

[48] Osawa Y Seki T Morita D. Total hip arthroplasty after transtrochanteric rotational osteotomy for osteonecrosis of the femoral head: a mean 10-year follow-up. J Arthroplasty. 2017;32:3088–92. 28634093 10.1016/j.arth.2017.05.020

[49] Kawano K Motomura G Ikemura S. Long-term hip survival and factors influencing patient-reported outcomes after transtrochanteric anterior rotational osteotomy for osteonecrosis of the femoral head: a minimum 10-year follow-up case series. Mod Rheumatol. 2020;30:184–90. 30556788 10.1080/14397595.2018.1558917

[50] Hasegawa Y Torii S Iwasada S. Pedicle bone grafting versus transtrochanteric rotational osteotomy for idiopathic osteonecrosis of the femoral head--four patients with both procedures. Nagoya J Med Sci. 1999;62:47–55. 10504827

[51] Sonoda K Yamamoto T Motomura G. Outcome of transtrochanteric rotational osteotomy for posttraumatic osteonecrosis of the femoral head with a mean follow-up of 12.3 years. Arch Orthop Trauma Surg. 2015;135:1257–63. 26173626 10.1007/s00402-015-2282-y

[52] Jacobs MA Hungerford DS Krackow KA. Intertrochanteric osteotomy for avascular necrosis of the femoral head. J Bone Joint Surg Br. 1989;71:200–4. 2925735 10.1302/0301-620X.71B2.2925735

[53] Kubo Y Yamamoto T Motomura G. Patient-reported outcomes of femoral osteotomy and total hip arthroplasty for osteonecrosis of the femoral head: a prospective case series study. SpringerPlus. 2016;5:1–8. 27833839 10.1186/s40064-016-3576-4 PMC5081314

[54] Osawa Y Seki T Okura T. Long-term outcomes of curved intertrochanteric varus osteotomy combined with bone impaction grafting for nontraumatic osteonecrosis of the femoral head. Bone Joint J. 2021;103:665–71. 33789486 10.1302/0301-620X.103B4.BJJ-2020-1107.R1 PMC8090999

[55] Schneider W Aigner N Pinggera O. Intertrochanteric osteotomy for avascular necrosis of the head of the femur. J Bone Joint Surg Br. 2002;84:817–24. 12211671 10.1302/0301-620x.84b6.12837

